# TE Density: a tool to investigate the biology of transposable elements

**DOI:** 10.1186/s13100-022-00264-4

**Published:** 2022-04-12

**Authors:** Scott J. Teresi, Michael B. Teresi, Patrick P. Edger

**Affiliations:** 1 Department of Horticulture, Michigan State University, East Lansing, Michigan USA; 2 Genetics and Genome Sciences Program, Michigan State University, East Lansing, Michigan USA; 3 Independent Researcher, Fredericksburg, VA USA

**Keywords:** Transposable Elements, Genomics, Genome Evolution, Bioinformatics, Python

## Abstract

**Background:**

Transposable elements (TEs) are powerful creators of genotypic and phenotypic diversity due to their inherent mutagenic capabilities and in this way they serve as a deep reservoir of sequences for genomic variation. As agents of genetic disruption, a TE’s potential to impact phenotype is partially a factor of its location in the genome. Previous research has shown TEs’ ability to impact the expression of neighboring genes, however our understanding of this trend is hampered by the exceptional amount of diversity in the TE world, and a lack of publicly available computational methods that quantify the presence of TEs relative to genes.

**Results:**

Here, we have developed a tool to more easily quantify TE presence relative to genes through the use of only a gene and TE annotation, yielding a new metric we call TE Density. Briefly defined as the proportion of TE-occupied base-pairs relative to a window-size of the genome. This new pipeline reports TE density for each gene in the genome, for each type descriptor of TE (order and superfamily), and for multiple positions and distances relative to the gene (upstream, intragenic, and downstream) over sliding, user-defined windows. In this way, we overcome previous limitations to the study of TE-gene relationships by focusing on *all* TE types present in the genome, utilizing flexible genomic distances for measurement, and reporting a TE presence metric for every gene in the genome.

**Conclusions:**

Together, this new tool opens up new avenues for studying TE-gene relationships, genome architecture, comparative genomics, and the tremendous diversity present of the TE world. TE Density is open-source and freely available at: https://github.com/sjteresi/TE_Density.

**Supplementary Information:**

The online version contains supplementary material available at (10.1186/s13100-022-00264-4).

## Background

Transposable elements (TEs) are mobile, repetitive DNA sequences that are major contributors to genome size and are found in almost every eukaryotic genome [1–4], with a possible exception being the protozoan *P. falciparum* [5]. Despite their ubiquity, they have historically been understudied and considered “junk” or “filler” DNA due to practical and theoretical reasons. Until recently, sequencing and assembling the repetitive portion of the genome was challenging and led to a lack of research within that section of the genome. Furthermore, the notion that the evolution of TEs is primarily shaped by their ability to replicate within a given host genome led researchers to overlook their capacity to create novel genotypic and phenotypic diversity, and thus contribute to adaptive evolution [6].

TEs also possess a rich taxonomic and phylogenetic history. This is best summarized by the sheer diversity of replication strategies, sequence structure, and genome distribution (reviewed in [7, 8]). At the most basic level, eukaryotic TEs can be broken into Class I and Class II elements based on their transposition mechanism and can be best summarized as “copy-and-paste” and “cut-and-paste”, although there are exceptions. Class I elements, also known as retrotransposons, utilize an RNA intermediate; whereas Class II elements, also known as DNA elements, utilize a DNA intermediate [7, 8]. Within each class, TEs can be further divided into order, superfamily, and family level descriptors. While a TEs’ class represents the presence or absence of an RNA transposition intermediate, a TE’s order represents major differences in insertion mechanism, organization, and enzymology, and the superfamily represents differences in protein-level and target site duplication (TSD) groupings. Finally, families represent commonalities in DNA sequence conservation.

TEs can impact the expression, directly or indirectly, of genes through a number of processes. For example, TEs can act as novel regulatory elements [9–16], promote alternative splicing [17], foster exon shuffling [18], duplicate nearby sequences (and sometimes entire genes) [19], influence ectopic recombination [20], create mutations through insertional mutagenesis [21–24], drive chromosomal rearrangements and gene transposition [25], promote sequence transduction [26], and become exons through exonization [27, 28].

As sources of mutation and genetic diversity, TEs are engaged in a number of interesting genotypes and phenotypes. In humans TEs are an active area of research as they are implicated in the development of cancer [23, 29–32]. They are also involved in a diverse set of congenital diseases such as hemophilia and cystic fibrosis to name a few [23, 33–37]. In animals, TEs have shaped peppered moth melanism [38], aided in the identification of subgenomes in the polyploid frog *Xenopus laevis* [39], evolved into circadian rhythm enhancers in mice [40], and were used to experimentally disrupt thousands of genes in *Drosophila* [41–43]. In plants, TEs have contributed to the wrinkled phenotype found in Mendel’s peas [44], grape color [45, 46], pepper disease resistance [47], apple color [48], wheat pathogen response [49], secondary metabolite variation in tomato [50], shaped coevolution between plants and microbes [51, 52], and are hypothesized to be associated with subgenome dominance in Monkeyflower and octoploid strawberry [53, 54].

The location of a TE profoundly influences its capacity to create variation. Generally, most TEs are located away from genes in the heterochromatic regions of the genome [55–58], and previous research has shown that gene expression is negatively correlated with TE presence [53, 59, 60]. TEs are transcriptionally silenced through a variety of mechanisms (reviewed in [61, 62]). However, sometimes the genes near TEs are also affected by this process, reducing their expression [60, 63–66]. This “collateral damage”, inflicted on genes via the spread of repressive chromatin marks associated with neighboring TEs, is an evolutionary trade-off that remains poorly understood. Exciting progress has been made in maize regarding the spread of methylation as it relates to different TE families but it remains unclear how generalizable these findings are between systems [63, 65, 67], especially when systems such as maize, rice, and *Arabidopsis* differ greatly in their TE content and epigenetic landscape. For example, TE-features associated with methylation spread in rice were ill-suited to predicting methylation spread in maize [65, 68].

The exceptional diversity of TEs within individual genomes and between genomes impedes the study of their effects on gene expression and genome evolution, which dovetails with a lack of standardized tools and approaches for analyzing TE presence relative to genes. Previous research has examined TE presence relative to genes but these methods have taken a path that is hard to compare and synthesize between systems. Biologically significant TEs are usually discovered and studied on a case by case basis; while bioinformatic approaches tend to focus on one specific type of TE, use a complicated TE presence metric, use inflexible genomic distances for measurement, and/or do not provide source code [30, 69].

Here, we present a new tool that enables the community to easily quantify TE presence relative to genes in any genome with an available gene and TE annotation. The TE Density tool calculates TE density values for all genes, upstream, intragenically, and downstream over any sequence of user-supplied windows. Our new metric, TE density, can briefly be defined as the summation of all TE-occupied base-pairs (for a given type of TE) taken in a measurement window (a discrete value of base-pairs, such as 500 base-pairs) relative to a given gene’s start or stop positions. See the implementation section (Implementation) for a more in-depth explanation of this metric.

Below, we provide five examples of how the tool may be used to investigate TE and gene biology. We utilize the human genome and a number of plant genomes as input datasets to illustrate the broad utility of the tool, and suggest potential applications of its output datasets. The analysis scripts for the examples and graphics described below are available along with the source code, and have been documented so that the user may easily utilize and expand off of them for their own research. First, we examine the average TE density of all genes as a function of window size and location in the *Arabidopsis thaliana* genome to describe general trends genome-wide. Second, we examine the relationship between gene expression and TE density in blueberry (*Vaccinium corymbosum*) as it changes according to TE type and position relative to a gene. We also examine the relationship between gene expression and TE density in Arabidopsis and how the status of a gene belonging to centromere/pericentromere or not impacts patterns. Third, we compare TE density between syntelogs of two closely related rice genomes (*Oryza glaberrima* and *Oryza sativa*) and show major TE differences amongst these positionally conserved genes. Fourth, we demonstrate how the tool may be used to quickly generate a summary table of TE density given a list of user-supplied genes; in particular, these genes are associated with cancer in humans when they are disrupted by TEs. Lastly, we show how TE density values may be used as a method to identify TE-impacted genes, potentially serving as a new procedure to generate lists of that could further analyzed by gene ontology (GO) enrichment analysis. We perform a (GO) enrichment on a set of exceptionally TE-dense genes and show that specific functional characteristics are underrepresented.

## Implementation

TE Density is open-source and freely available at: https://github.com/sjteresi/TE_Density. The code used to analyze and create the figures for the usage and application examples shown in this manuscript is also freely available within the code repository. Users are encouraged to re-use and extend that code for their own analyses. This section includes the usage, design, and implementation of the toolkit. Users are encouraged to visit the project’s GitHub README for more of an in-depth description of usage.

### Design

The goal is to calculate TE density for the combination of (*superfamily* ∥ *order*)×(*left* ∥ *intra* ∥ *right*), with respect to a window length and an individual gene. The output matrices, representing the TE density data for each pseudomolecule, are of size |*identity*|×|*windows*|×|*genes*|×|*direction*|, where *identity* is the set of either the TE superfamilies or orders, *windows* is the set of window lengths, and *genes* is the set individual gene names. The *direction* is the relative location of the window to a gene’s start and stop position, where *direction*∈*left*, *intra*, *right*. Since genes are typically organized within annotation files with their *start* position as the least-greatest base-pair value, regardless of whether or not the gene is in an antisense or sense facing direction, we chose to use the terms “left” and “right” during development, as that more appropriately corresponds to increasing or decreasing base-pair values. *Left* corresponds to a window sweep of base-pairs less than the gene’s start position, *intra* between a gene’s start and stop position, and *right* for a sweep greater than the gene’s stop position. The left and right directions are later converted during postprocessing to correctly correspond to upstream and downstream, discussed in 3. The knowns are the start / stop locations of the TEs and genes, the names of the genes, and the superfamily / order identity of the TEs. The problem is simplified by splitting each density calculation with respect to each pseudomolecule, as there can be no overlap of TEs and genes between said pseudomolecules.

The main software design goal was to allow for the analysis of other larger genomes which translated to improving testability and execution speed. Improving the testability makes the toolkit easier to use and simplifies the data cleaning stage. Improving speed expands the number of genomes that would be feasibly analyzed by reducing the time required to obtain results. This speed was achieved through numpy best practices, see Performance for a discussion. The package was tested on a CPython implementation of Python 3.8.0. The standard requirements.txt file is within the requirements directory and the package is named transposon.

### Pipeline

See process_genome.py -h for the main entry point and description. This calculates density and writes the results to disk for later analysis. Callers must clean and reformat their annotation files as described in 3 and the README. These reformatted annotation files are the primary inputs to process_genome.py. Cleaning the data should be sufficient, however for a more complete demonstration of usage please refer to the examples directory and 3.

**Fig. 1:**
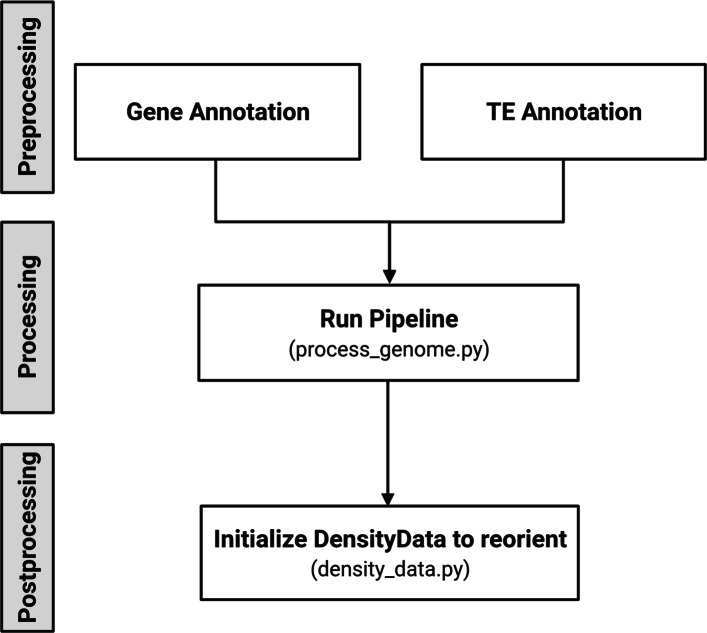
Flowchart of the TE Density pipeline

The data pipeline stages are: *preprocessing*, *processing* (overlap, summation), and *post-processing* as seen in Fig. 1. Preprocessing reformats and cleans the input gene and TE annotations for downstream computation. The processing stage begins with calculating TE overlap within the search window and its sum. The overlap outputs are the number of base pairs occupied by the transposable element within a window offset from the gene. Overlaps are summed across the identity of the TE, which is the superfamily or order identity for this stage. This sum is then normalized according to the search window. Finally, the post-processing stage modifies the left and right direction values to better correspond to sense and antisense gene orientations of upstream and downstream. We will discuss the stages of the pipeline in order of operation.

#### Preprocessing

The preprocessing stage is responsible for reformatting and cleaning the input data. It requires two principal input data files: a gene annotation and a TE annotation. The user must minimally reformat each annotation for usage in the pipeline; this corresponds to the “Gene Annotation Filtration” and “TE Annotation Filtration” portions of Fig. 1.

We provide scripts and guides within the project README to accomplish this. During this stage the user may reclassify or omit TE groupings (orders and superfamilies) found in the TE annotation file. For example, the user may want to perform a simple rename of a TE grouping such as changing “EnSpm_Cacta” to “CACTA”, change the main grouping of a TE to its own independent grouping, or merge it into another grouping. During the preprocessing of the Arabidopsis dataset we redefined *LINE/Penelope* elements into their own order of *PLE/Penelope* in order to correspond to the classification scheme proposed by Wicker et al. ([7]) and better reflect the differences of the two TE groupings.

**Annotation Revision** One preprocessing activity of particular importance is revision where TEs are “revised” to eliminate overlapping TEs. During development, we found that TEs can frequently overlap with other TEs in a given TE annotation. This could either be through TEs inserting within other TEs or due to artifacts arising from the annotation software. Since TE density is defined as the total number of TE-owned base-pairs divided by the relevant base-pair window, overlapping TEs would lead to some base-pairs being double-counted. If you assume that each base-pair in a given window can only be occupied by one entity (gene, TE or other), this double-counting violates that assumption, and would inflate TE density values past 100% density. While some of these TEs may be biologically real, we chose to modify the TE annotation in a preprocessing manner rather than discard overlapping TEs and lose data. Thus, in order to avoid creating this math and interpretation error without compromising our ability to quantify TE presence, we merge the positions of similar (using the TE’s order and superfamily identities) TEs prior to computation of TE density in order to simplify the mathematics of our pipeline. Below, we describe the merging process in more detail.

Briefly, overlapping TEs in the annotation are condensed into a singular TE if their identities match. For example, when calculating superfamily values if two individual TEs, both of the LTR/Copia type, appear in the dataset with partially overlapping positions, we merge them into one contiguous TE by redefining the start and stop positions of the TEs. Please see Supplemental filesTest_SingleC_SingleElongate_Superfam_Re-vision.tsv and SingleC_SingleE_Super.tsv for one example of the input and output of the revision process. Below we describe the revision process in more detail.

**Dealing with Overlapping TEs** The revision process is done on a separate basis for all order, and superfamily groupings. It is performed a third time for all TEs and given the grouping “Total TE Density” so that we can accurately calculate total TE density irrespective of TE groupings. As previously stated, the process takes place in 3 independent steps: first, only TEs of the same Order grouping are merged, second only TEs of the same Superfamily grouping are merged, third all TEs are merged together to create a new entry with the “Total TE Density” grouping. For each revision process the original TE annotation is broken into subsets which are comprised of the same TE grouping. This subset is then searched recursively one entry at a time in order to locate all possible merges for the seed TE. Candidate TEs are merged together resulting in an intermediate dataframe comprised of non-overlapping TEs, all of the same grouping. Once the search space is exhausted, the code moves on to the next entry (TE) and the process begins anew. Once this process is completed for all TE identities, the dataframes are concatenated into the resultant revised annotation. A dummy category is introduced during the merging operation to distinguish which grouping is being actively merged, hence the usage of S_Revision in the files provided. This dummy category of TE density can later be discarded during postprocessing.

Overall the revision process method creates at maximum three entries (individual TEs) for every single entry in the original annotation; the first would be a TE signifying any relevant merges along the Order identity, the second would represent any relevant merges along the Superfamily identity, and the third would represent a merged TE resulting from any other overlapping TEs regardless of identity. An individual TE may result in less than the maximum of 3 new entries if it happens to merge with another TE along a certain grouping. The resulting “revised” TE annotation, allows the pipeline to accurately calculate TE Density for all TE groupings and for the total TE Density category while keeping values bounded between 0 and 1.

#### Processing

Overlap for *left* (*O*_*l*_), *intra* (*O*_*i*_), *right* (*O*_*r*_) are shown in (1) (2) (3) respectively. The inputs are *w*=*windowSize*,*g*_0_=*geneStart*,*g*_1_=*geneStop*,*t*_0_=*transposonStart*, and *t*_1_=*transposonStop*. The overlap is simple albeit verbose in order to account for the different directions whilst clipping the bounds accordingly. The *intra* overlap is a special case that is not swept with respect to the window but instead the bounds of the gene.

1 $$ \begin{aligned} w_{0} &= \max(w_{1}- w, \; 0)\\ w_{1} &= g_{0} - 1\\ b_{0} &= \max(w_{0}, \; t_{0})\\ b_{1} &= \min(w_{1}, \; t_{1})\\ O_{l} &= \max(0, \; (b_{1} - b_{0} + 1)) \end{aligned}  $$

2 $$ \begin{aligned} b_{0} &= \min(g_{0}, \; t_{0})\\ b_{1} &= \max(g_{1}, \; t_{1})\\ O_{i} &= \max(0, \; (b_{1} - b_{0} + 1)) \end{aligned}  $$

3 $$  \begin{aligned} w_{0} &= g_{1} + 1\\ w_{1} &= w_{0} + w\\ b_{0} &= \max(w_{0}, \; t_{0})\\ b_{1} &= \min(w_{1}, \; t_{1})\\ O_{r} &= \max(0, \; (b_{1} - b_{0} + 1)) \end{aligned}  $$

*Left* and *right* density (*ρ*_*l*, *r*_) is shown in (4), and *intra* (*ρ*_*i*_) in (5). These are generalized for one *direction*, *window*, *gene*, and *identity*. The subscript (*i*) is the index of said TE identity, such as the superfamily or order for this analysis. Note that the density *ρ*_*l*_ and *ρ*_*r*_ is normalized by *w*+1 and not *w*. This is because the search window [*w*_0_…*w*_1_] for calculating the bounds is offset by one in *w*_0_,*w*_1_ of (1) (3). Note also that the *intra* density is normalized by the element count of the gene in question, which is *g*_1_−*g*_0_+1 as the elements are zero indexed and inclusive on both sides.

4 $$ \begin{aligned} \rho_{l, \;r} = \frac{1}{w+1} \sum_{i=0}^{N-1} O \end{aligned}  $$

5 $$  \begin{aligned} \rho_{i} = \frac{1}{g_{1} - g_{0} + 1} \sum_{i=0}^{N-1} O \end{aligned}  $$

Algorithm 1 shows pseudo-code for the overlap and summation stages. It is simplified for one pseudomolecule as each are independent. The directions *left*, *intra*, *right* are omitted for brevity. The overlap calculation is essentially a subtraction between the bounds shown in (1) (2) (3) applied for all genes and TEs, and swept over the windows. The density calculation is essentially a sum over the overlap results that are indexed with respect to the *identity*, also swept over the windows.


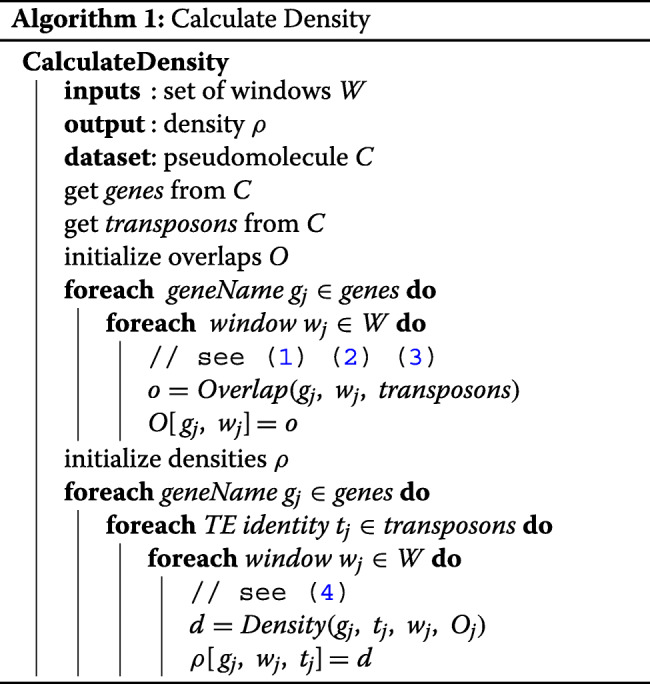


Processing the genome yields a density file for each pseudomolecule formatted in HDF5. Each represents the TE density values for an individual pseudomolecule in the annotation. The output densities have the dimension |*identities*|×|*windows*|×|*genes*|×|*direction*|. The DensityData class is used in postprocessing and provides access to the sub-arrays of the result and generates the tables shown in Table 2 and Supplemental Table S1.

#### Post processing

Post-processing, the left and right density values of anti-sense genes are swapped to accurately correspond to the traditional upstream and downstream descriptions of a gene. The *DensityData* class performs this step upon initialization. As previously stated, due to the convention that start and stop positions are presented in annotation files with the start position always being less than the stop position, even if the gene is in the antisense orientation, we chose to use the *left*, *intra*, *right* terminology in the implementation of the pipeline.

### Testing

The pipeline was tested with pytest to verify the mathematics and system. Tests include but are not limited to: the creation of the revised TE annotations, the calculation of TE base-pairs within a window (overlap and summation), the importation of gene and TE annotations, and the calculation of density. One may run the tests like so in the project root directory: python3 -m pytest.

### Performance

Performance was a high risk at the start of this work as analysis of large genomes might not be feasible if the execution time is too long, i.e. days or weeks. One reason for this is that this work contains the inner loop problem as seen in Algorithm 1. This is exacerbated as Python can be particularly susceptible to it, and the calculations also have to be repeated for *left* / *right* directions as well as the *superfamily* / *order* identity. Thankfully this risk was mitigated using multiprocessing and numpy vectorization and a formal optimization was not necessary. While this work is not optimized nor should it necessarily be used as a canonical example, it is worth noting that there are simple steps one can take to achieve reasonable results. Multiprocessing was an easy first solution as each pseudomolecule (chromosome) is independent. Vectorization in numpy was important for calculating the overlap bounds as well as summing the overlaps given a matching TE *identity*. Finally, HDF5 file format was chosen for efficient *I*/*O* to the output density files. Future work may consider fleshing out profiling, using a split / merge pattern, and / or numba.

Table 1 displays the performance metrics for the genomes used example applications in this paper. Factors such as the number of windows, the number of unique TE order groupings, and the number of unique TE superfamily groupings likely have the most impact on the performance of the tool, as they each add an array to the output. Notably, the human example took a relatively long time when compared to the other genomes present. We suspect that the number of TE calculations, which increases with additional unique TE groupings, is the primary factor in its increased computation time. For example, there were many TEs with family-level identities being treated as individual superfamilies, such as “hAT-Ac”, “hAT-Blackjack”, “hAT-Charlie”, “hAT-Tag1”, “hAT-Tip100” etc, that likely could have been condensed into the already present “hAT” group. Simple steps to mitigate this issue could include condensing TEs of similar groups into a singular group, as described in 3. This would reduce the amount of calculations needed, speed up the tool, and arguably simplify downstream analyses.

**Table 1:** Table of tool performance. Performance was estimated using an Intel Xeon CPU E5-2670 v2 possessing a processor base frequency of 2.50 GHz. Statistics were acquired using the “seff” command on the SLURM workload manager for the computing cluster at Michigan State University. TE Density calculations are performed over chromosomes (pseudomolecules) independently, each chromosome can only utilize one processor at a time. * Chromosomes 7 and 13 were used for the human genome dataset, the genome size is the sum of the lengths of the two chromosomes. The repeat content was not reassessed on a chromosome-by-chromosome basis, and the percentage is referenced from publications [70]. *†* For the other genomes, repeat annotations and content estimates were derived using the de-novo TE annotator EDTA and may differ from each genome’s respective datasets. Wall time and CPU hours are in day-hh:mm:ss format. Default windows were used (n=20) and the variable, “Max TE Calculations” is defined as the maximum number of unique TE order or superfamily names, whichever is greater

Genome	Chromosome Number	Processors Used	Genome Size (GB)	Repeat Content	Wall Time	CPU Hours	Memory Utilized (GB)	Window X Max TE Calculations
*A. thaliana*	5	5	∼0.135	14.91% †	00:51:31	02:32:16	32.18	20 X 13
*V. corymbosum*	48	20	∼1.630	46.50% †	19:34:19	1-23:02:32	603.77	20 X 14
*O. sativa*	12	12	∼0.500	49.46% †	03:17:23	07:20:49	243.54	20 X 13
*O. glaberrima*	12	12	∼0.358	39.90% †	02:15:09	04:53:56	154.18	20 X 13
*H. sapiens*	2*	2	∼0.274*	66% *	5-22:20:15	6-00:07:52	164.80	20 X 35

In the interest of completeness, we provide the memory utilized statistic for each genome in Table 1, however the statistics may be misleading due to the fact that our goal was to run each genome through the complete pipeline as fast as possible. This mean that we used as much memory (RAM) and processors as we could reasonably request. In practice, users may have less processors and less RAM. Additionally, users only need to generate the revised annotation, as described in Preprocessing section, once; however this calculation was included in the time estimates for Table 1, and inflates the time needed. This will save time if users plan to generate TE Density data more than once, for example users may wish to generate data for a different set of windows than the ones initially used.

### Examples

Version controlled documentation and code related to recreating each analysis can be located in the project GitHub repository within the examples/ directory. There is a Makefile within each genome’s directory that can be used as a reference to see how analyses and scripts were executed.

### Repeat annotations

EDTA was used to generate a TE annotation for the *Arabidopsis thaliana*, *Vaccinium corymbosum*, *Oryza sativa*, and *Oryza glaberrima* genomes [71]. The scripts for recreating each genome’s EDTA annotation can be found within its respective src/ directory within the examples directory. EDTA was run with a genome FASTA file and a CDS FASTA file. For each genome other than *Vaccinium corymbosum*, a CDS FASTA file was created using GFFRead version 0.12.6 [72]. Default options were used for EDTA in all cases except for the usage of the –cds, –sensitive 1, and –anno options. The –sensitive 1 option tells the program to use RepeatModeler to identify remaining TEs that were missed by structure-based methods following the normal progression of the pipeline [73]. Following the creation of the EDTA annotation, a custom script was used to modify some of the TE groupings in order to reorganize and conform to the naming system presented in [7] for simplicity in analysis. An example of this script can be found in examples/Arabidopsis/src/replace_names_Arabidopsis.py.

The *Homo sapiens* repeat annotation was downloaded from https://genome-euro.ucsc.edu/cgi-bin/hgTables and filtered into a TE Density-appropriate format with src/import_human_te_anno.py. After importing the TE information, a custom script was used to modify some of the TE groupings in order to remove low confidence entries, reorganize and conform to the naming system presented in [7] for simplicity in analysis, which can be found in examples/Human/src/replace_human_TE_names.py, some TE groupings were left intact in order to assess pipeline performance on a genome with a large amount of groupings.

For all genomes, TE Density was run with default options, yielding an HDF5 file for each chromosome containing TE density values for each gene, for each window, for each TE order, and for TE superfamily.

### Arabidopsis example methods

The Arabidopsis genome and gene annotation files were taken from TAIR V10 [74]. Total RNA was extracted from fresh young leaf tissue using the Invitrogen PureLink RNA Mini Kit, converted into an Illumina library using the TruSeq RNA kit (Illumina), and paired-end 100-bp reads were sequenced on the HiSeq- 2000 instrument at the University of Missouri DNA core. The NextGENe V2.17 (SoftGenetics, State College, PA, USA) software package was used to remove low-quality data, aligned to the Arabidopsis thaliana TAIR10 genome [74], and FPKM (fragments per kilobase million) normalized.

The src/compare_centromeric_densities.py script was used to analyze the relationship between TE density and a gene’s location within or outside of the pericentromere. A gene’s status of belonging to centromere or pericentromere was assessed using data from Colomé-Tatche et al. [75]. The src/generate_dotplots.py script was used to analyze average TE Density values of genes as window size increases.

### Blueberry example methods

The blueberry genome and gene expression datasets were derived from [76]. The Vaccinium_corymbosum.faa FASTA file and Vacc_c_CoGe_CDS.fasta CDS FASTA file were downloaded from CoGe [77], and used as primary inputs to the EDTA pipeline. The src/compare_expression.py script was then used to analyze the relationship between gene expression and TE density.

### Rice example methods

Rice FASTA files and gene annotation were derived from the https://ensemblgenomes.org/ website. The Release 50 version was used for both *Oryza sativa* and *Oryza glaberrima* [78]. Each genome was uploaded to https://genomevolution.org/coge/ and the https://genomevolution.org/coge/SynMap.pl tool was used to identify syntelogs between the two genomes [79]. The analysis can be replicated using the following link https://genomevolution.org/r/1how2. The syntelogs were then filtered using the import_syntelogs.py script which primarily filtered out any pairs with an E-value greater than 0.05. The src/compare_density.py script was then used to analyze TE density differences among the syntelog pairs. Applying a percentile cutoff and identifying the genes that met the cutoff was done within the src/find_abnormal_genes.py. Once a TE density percentile cutoff value was determined and an array of genes was created, we ran the genes through PANTHER [80, 81].

### Human example methods

Human datasets (Version 38) other than the TE annotation were derived from the UCSC Genome Browser at http://hgdownload.soe.ucsc.edu/goldenPath/hg38/ [82].

The info_of_gene method in the DensityData class was used to generate the tables for the BRCA2 and CFTR genes.

## Results

### TE density can reveal underlying trends of how TEs are positioned relative to genes

**Fig. 2:**
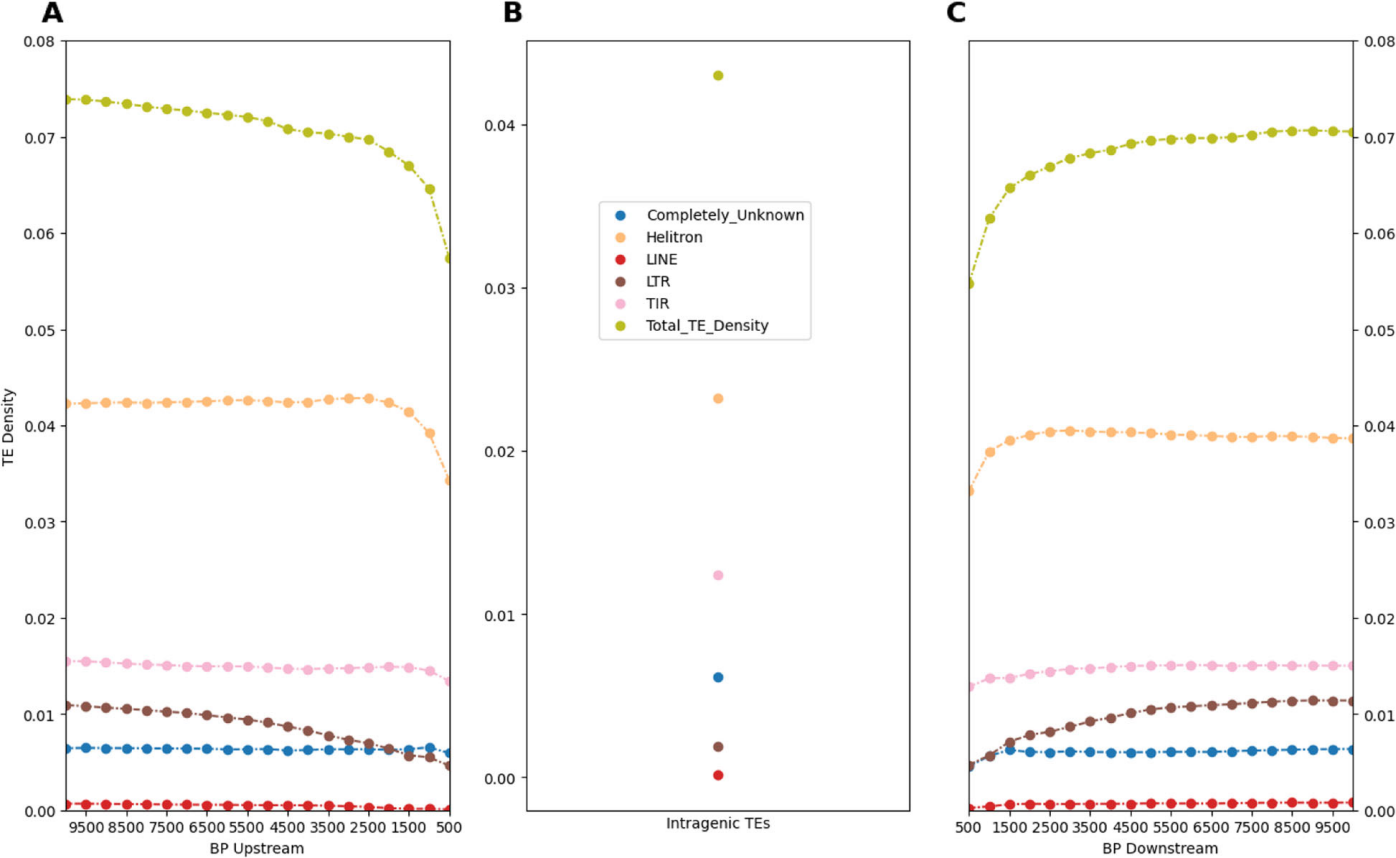
Average TE Density of All Genes as a Function of Window Size and Location: Data represents average TE Density values for all genes for a given window and direction for Arabidopsis chromosome 1. Panel **A** represents the TE density values upstream of genes, panel **B** represents the intragenic TE density of genes, and panel **C** represents the TE density values downstream of genes

The TE Density tool’s data and associated analysis scripts can reveal small and large scale patterns of transposon presence. We ran the Arabidopsis genome through the TE Density tool and created Fig. 2 to show the average TE density values, using the TEs’ order identity, for every gene in chromosome 1 as they correspond to the various TE types, and how these values change as the measurement distance relative to the gene increases. Figure 2, parts A and C, show one way in which the TE Density data may be used to examine genome-wide trends of TE positioning relative to genes, and interrogate upstream versus downstream differences. Figure 2 part B shows how intragenic TE density may be examined.

For example, Fig. 2A shows greater average upstream Helitron TE density values than downstream; the total TE density metric replicates this as well. However these observed density differences in upstream and downstream values are not significantly different based on a chi-square test (*χ*^2^ = 0.0056; *p*-value ≥0.9). Upstream LTR TE density values are lower than completely unknown TEs (TEs’ whose order and superfamily identities were unable to be determined) for small window sizes, but they are greater than the unknown TEs by the 2 KB window. However, this trend is different when considering downstream values, both groupings start out at very similar levels, but LTR elements quickly overtake completely unknown TEs to occupy a greater share of base-pairs by the 1.5 KB window. Similar to Helitron TEs, these observed differences in up and downstream density values for LTR elements are not significantly different based on a chi-square test (*χ*^2^ = 0.3347; *p*-value ≥0.5). The intragenic subplot (Fig. 2B) generally replicates the upstream and downstream trends, in that each TE type maintains its relative position in density values compared to other the TE types. However, there is one exception, genes have a higher average intragenic density for completely unknown TEs than they do for LTR TEs. One possible explanation for this phenomenon is that the completely unknown TEs represent TEs that are decayed and inactive, and that the intragenic space possesses a higher share of these TE “graveyards” than the upstream and downstream locations, due to a greater force of selection purging TEs in close proximity to exons.

### TE density and its relation with gene expression

The TE Density tool can easily be used in conjunction with gene expression data to investigate the relationship between TE presence and gene expression. Here, we examine how gene expression profiles change as TE density increases or decreases. Using previously published gene expression data from the high-bush blueberry *Vaccinium corymbosum* genome [76], we plotted gene expression values as a function of binned TE density values for all non-lowly expressed genes in the genome. Similarly, we plot the expression values of *Arabidopsis thaliana* genes as a function of binned TE density values while distinguishing between those belonging to the centromeric/pericentromeric region and those that do not. These data are further discussed below.

**Fig. 3:**
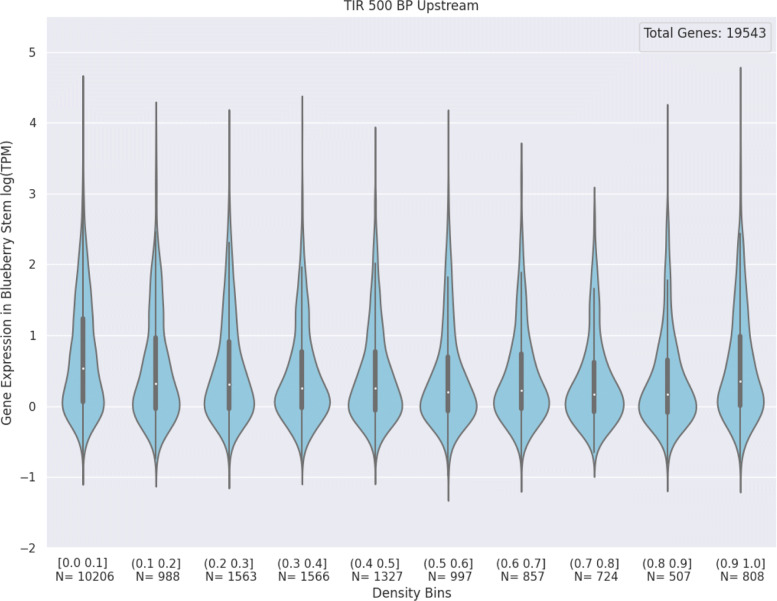
Violin Plots of TE Density vs Gene Expression: Density values are derived from the TIR TE grouping for the 500 BP window upstream of each gene. Underneath each violin plot is the interval of TE density values that bins the genes being plotted. Underneath each density bin is N, the number of genes for that given bin. Lowly expressed genes, genes with less than 0.1 TPM, were excluded from the plot. The solid dark band inside each violin represents the interquartile range (IQR) of the expression values. The white dot inside the IQR represents the median. The “whiskers” extend 1.5x past the IQR in both directions

**Expression Profiles of Genes with High Density Are Not Too Dissimilar From Low Density Genes** Figure 3 shows how the number of genes and their expression profiles change as TE density increases. Generally, the expression profiles of the high density genes are similar to the low density genes, as the median expression and inter-quartile range are roughly similar, however the high density bins tend to have fewer genes. As window size increases the number of genes decreases and their range of expression values becomes more constrained. This trend is best shown when examining the Copia 500 BP and Copia 10 KB plots (Supplemental Figs. S1 and S2).

**Fig. 4:**
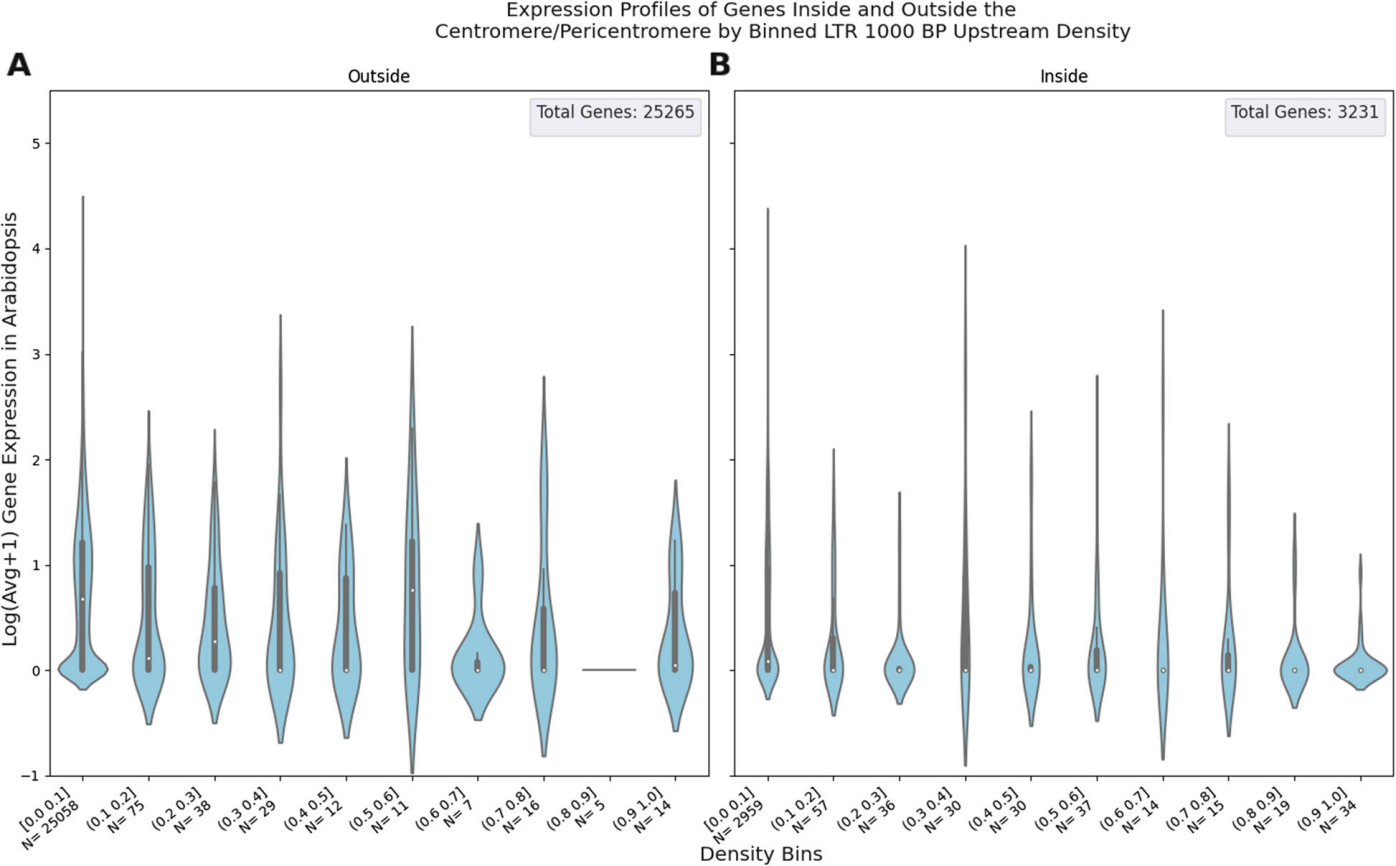
Violin Plots of TE Density vs Gene Expression in Arabidopsis: Density values are derived from the LTR TE grouping for the 1000 BP window upstream of each gene. Underneath each violin plot is the interval of TE density values that bins the genes being plotted. Underneath each density bin is N, the number of genes for that given bin. The solid dark band inside each violin represents the interquartile range (IQR) of the expression values. The white dot inside the IQR represents the median. The “whiskers” extend 1.5x past the IQR in both directions. Subplot **A** represents the genes that *do not* belong to the centromere or pericentromere. Subplot **B** represents the genes that *do* belong the pericentromere or centromere

We also examined the expression profiles of genes binned by TE density while distinguishing between genes that reside in the centromere/pericentromere and those outside in Fig. 4. The genes inside the centromere/pericentromere sometimes have a more constrained range of expression values and generally have more genes with greater TE density. The increased number of genes in more dense bins may be better be visualized in Supplemental Fig. S3.

**The Number of Expressed Genes Generally Decreases as TE Density Increases** Interestingly, the number of genes in a given TE density bin does not consistently decrease as TE density increases. Figure 3 demonstrates this trend well, there is a local maxima in the number of genes per bin for the (0.2,0.3] interval of density values.

**Fig. 5:**
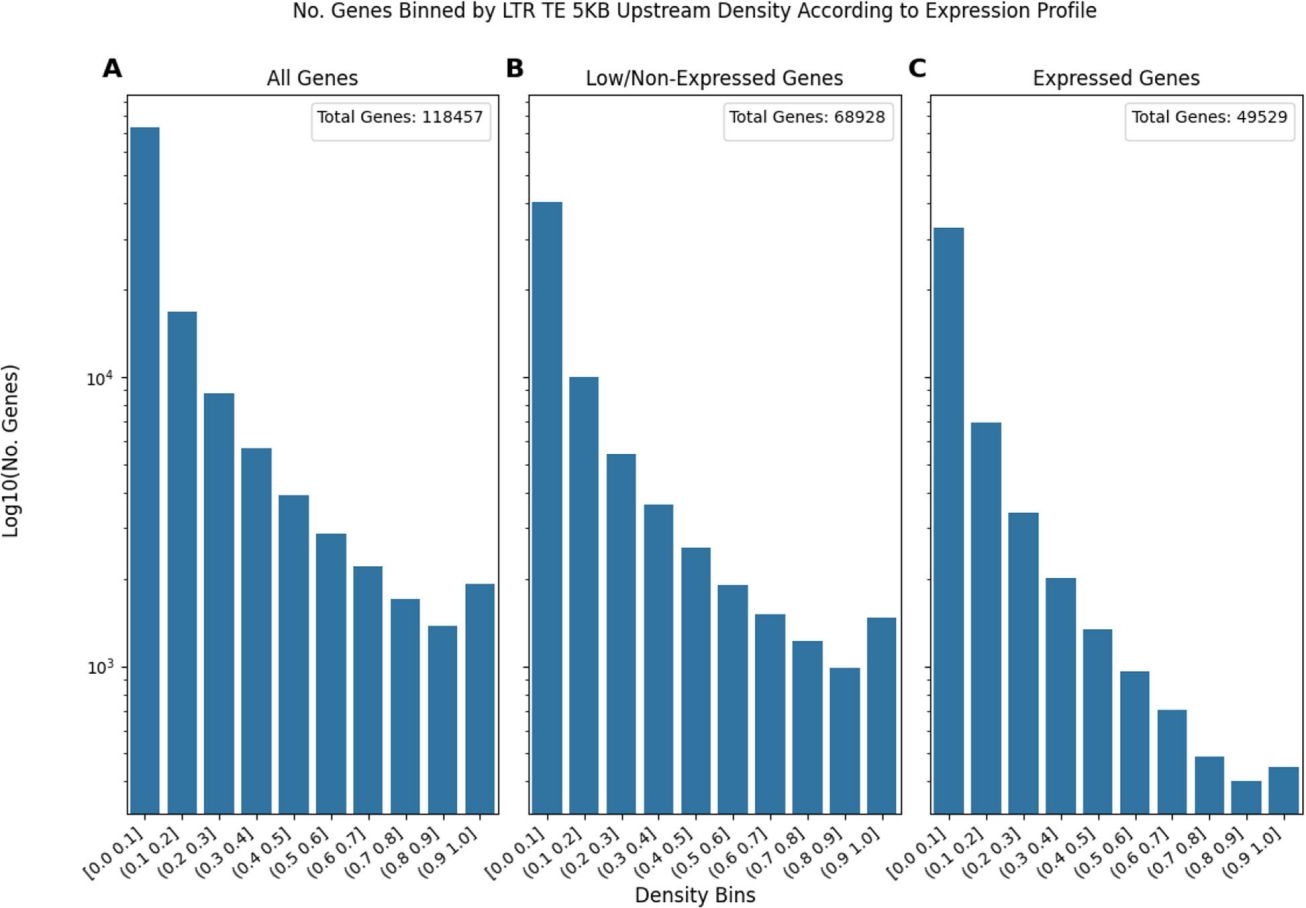
Bar plots of the number of genes in a given TE density bin as TE density increases. The density of LTR elements 5KB upstream of genes is shown. The number of genes was log10(x+1) transformed to better display the y-axis data. Subplot **A** represents the number of genes in each density bin for all genes. Subplot **B** represents the genes that are lowly expressed (less than 0.1 TPM) or non-expressed (0 TPM). Subplot **C** represents the genes that are expressed (expression greater than or equal to 0.1 TPM)

**Fig. 6:**
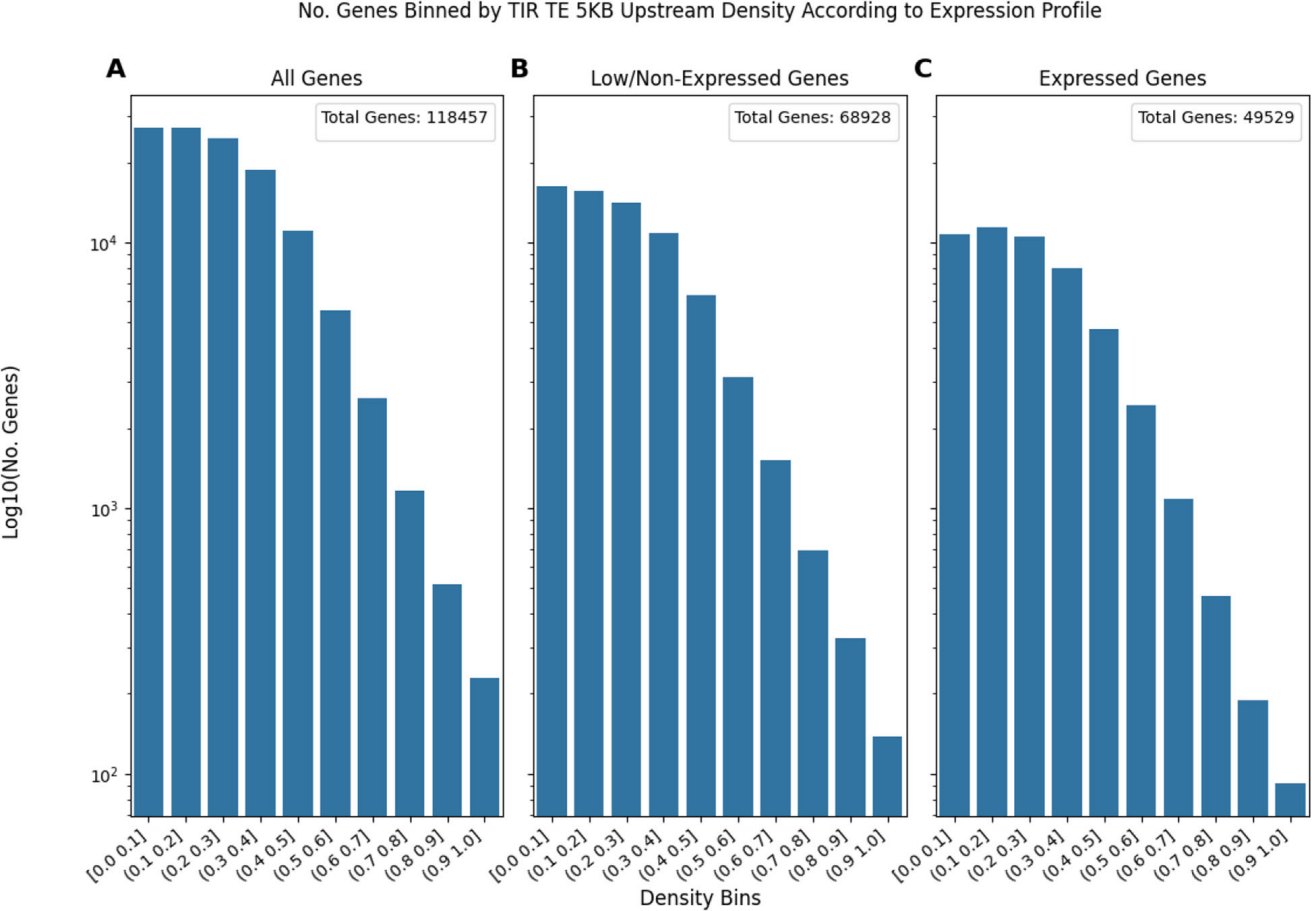
Bar plots of the number of genes in a given TE density bin as TE density increases. The density of TIR elements 5KB upstream of genes is shown. The number of genes was log10(x+1) transformed to better display the y-axis data. Subplot **A** represents the number of genes in each density bin for all genes. Subplot **B** represents the genes that are lowly expressed (less than 0.1 TPM) or non-expressed (0 TPM). Subplot **C** represents the genes that are expressed (expression greater than or equal to 0.1 TPM)

**The Number of Genes in Each TE Density Bin Decreases Differently When Comparing Different TE types** Comparing Figs. 5 (LTR elements) and 6 (TIR elements) demonstrates this trend. In Fig. 5 the number of expressed genes consistently decreases as TE density increases, save for a tiny local maxima at the most dense bin of (0.9,1.0]. On the other hand, Fig. 6 shows how the number of expressed genes remains relatively stable for about the first 5 bins before the number of genes starts to drop. Taken together, Figs. 5 and 6 suggest that there are transposon presence patterns that are not necessarily affected by gene expression status.

### Syntelog TE density differences (Rice)

The TE Density tool can also be used to compare TE presence values between genes of different genomes. In this way, the tool may be used to examine presence-absence variation of TEs between genomes, and used as a screen to identify potentially TE-impacted genes. Pangenome analyses have largely focused on gene-space differences and have generally outpaced the analysis of the TE-space. This tool allows for a reproducible comparison of gene-centric TE-variation amongst genomes. Here, we compared TE levels of syntelogs belonging to two closely-related rice genomes, *Oryza glaberrima* and *Oryza sativa*, and found major differences in TE density values. We calculated the difference in TE density values on a syntelog-pair basis and found that values were as great as |1.00|, suggesting complete presence/absence variation of TEs.

**Fig. 7:**
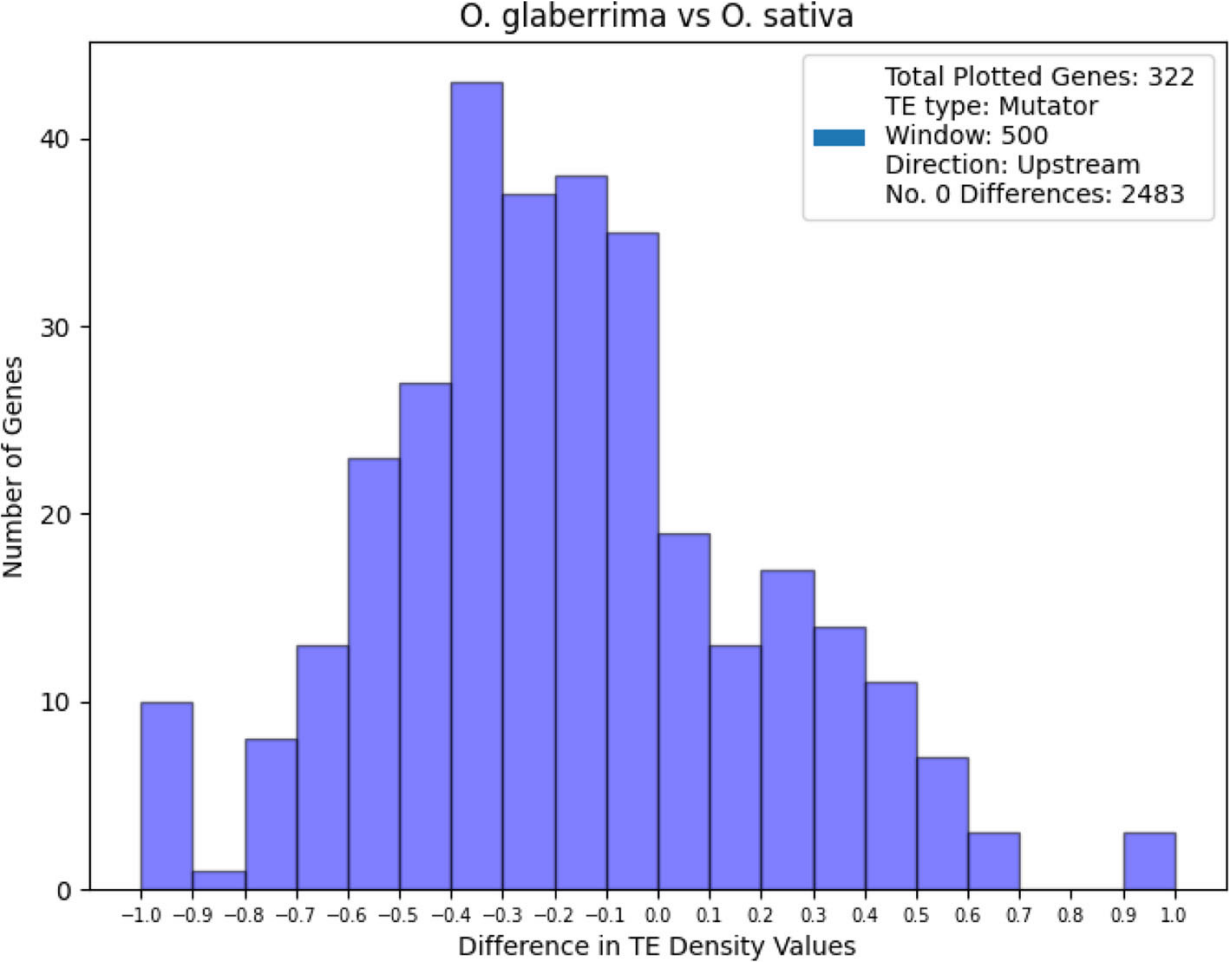
Histogram of differences in TE Density values of syntelogs of chromosome 1 for *Oryza sativa* and *Oryza glaberrima*. The density values for the TE type being shown are derived from the Mutator superfamily grouping of TEs, and values were collected in a 500 bp window upstream of genes. TE Density difference values were calculated by subtracting *Oryza sativa* values from *Oryza glaberrima* values. Negative values reflect a higher TE value for the *Oryza sativa* syntelog and positive values reflect a higher TE value for the *Oryza glaberrima* syntelog. Values were binned into groupings reflecting 10% increases or decreases in TE Density. All but the last (most positive) bin is half-open. For example, the leftmost bin reflects an interval of [−1.0,−0.9) and the rightmost bin reflects an interval of [0.9,1.0]. The number of syntelog pairs shown in the histogram is 322; the number of syntelog pairs without any difference in TE density values is 2483

Figure 7 shows a histogram of these differences in TE density values. Here, the TE density differences were calculated using the Mutator TE grouping, the 500 bp upstream window, and genes derived from chromosome 1 of the two rice genomes. Interestingly Fig. 7 shows a general greater TE density surrounding the *Oryza sativa* syntelog compared to the *Oryza glaberrima* syntelog.

Previous work in *Arabidopsis* highlights interesting trends in TE and gene expression divergence between closely related species; comparing *Arabidopsis thaliana* and *Arabidopsis lyrata*, Hollister et al. showed that orthologs possessing zero TEs within 1KB did not differ significantly in expression, but when both orthologs had any TE within 1KB the *A. thaliana* copy was significantly lower expressed [83]. They also showed that when only one ortholog had any TE the expression divergence was significant only if the TE was targeted by siRNAs [83]. The TE Density tool provides an easy-to-use, reproducible platform to further explore the effect of TEs on divergent gene expression in a diverse set of genomes, using specific TE groupings and a finer-scale system of measurement.

### Human genome interesting genes and their TE density levels

As previously mentioned, the TE Density tool and analysis scripts can be used to calculate and inspect the TE presence values of specific genes; in this case the tool was used to quantify and explore the TE levels of genes that are known to cause various diseases in humans when disrupted by TEs, such as cancer, reviewed in [84]. In the near future as personalized medicine will likely generate reference genomes for individual patients, this tool could be used for screening TE variants distributed across the genome. The TE Density tool contains a convenient analysis script that produces a summary table for each gene in a user supplied list of genes which reports the greatest and least dense TE groupings along with their values. Here, we used that script to generate Table 2 which represents the TE density information of the BRCA2 gene.

**Table 2:** Table of greatest TE density values by TE type for the BRCA2 gene in a 1 KB window upstream and downstream. SINE elements occupy ∼40% of the 1000bp window upstream, examining the Superfamily section reveals that it can further be broken down into MIR and Alu elements with a ∼10% and ∼30% share, respectively. Intragenically, LINE and SINE elements contribute to the greatest share of TE Density

Top 5 TE Orders					
Upstream:					
Identity	No TE	No TE	No TE	SINE	Total_TE_Density
Density	0	0	0	0.401	0.401
Intragenic:					
Identity	LTR	TIR	SINE	LINE	Total_TE_Density
Value	0.032	0.073	0.194	0.226	0.524
Downstream:					
Identity	No TE	No TE	No TE	No TE	No TE
Value	0	0	0	0	0
Top 5 TE Superfamilies					
Upstream:					
Identity	No TE	No TE	MIR	Alu	Total_TE_Density
Density	0	0	0.1	0.301	0.401
Intragenic:					
Identity	TcMar-Tigger	L2	L1	Alu	Total_TE_Density
Value	0.042	0.091	0.132	0.171	0.524
Downstream:					
Identity	No TE	No TE	No TE	No TE	No TE
Value	0	0	0	0	0

#### Inspection of the BRCA2 gene

The BRCA1 and BRCA2 genes, are best known as breast cancer susceptibility genes [34, 37, 85, 86]. It has previously been shown that breast cancer can be caused by genomic rearrangements of the BRCA genes [37], and that transposable elements may sometimes be the culprit. In some cases, these genomic rearrangements can lead to exon skipping, and Alu insertions near BRCA2 have been implicated in exon skipping [34, 37, 86]. Interestingly, the BRCA1 gene’s intronic regions are also known to be rich in Alu sequences [87].

Table 2 represents the output of the analysis script. It displays the TE levels surrounding the BRCA2 gene in the human reference annotation and may be used as a quick diagnostic to see if a locus has changed from an expected value. It appears that the BRCA2 gene has 0 TE presence 1KB downstream, but has relatively high (0.401) upstream SINE density. Its intragenic density is an amalgamation of multiple TE types with the SINE and LINE categories taking up most of the space.

On a similar note, we investigated the CFTR gene, also known as the cystic fibrosis transmembrane conductance regulator gene. It can also be affected by aberrant Alu element insertions, giving rise to cystic fibrosis in affected individuals [35]. Supplementary Table S1 displays the TE levels surrounding the CFTR gene in the human reference annotation. The goal of this section is to highlight that the tool is capable of inspecting TE density of target genes. Here we showcase two genetic variants with known TE insertions that are associated with a human disease trait. The tool could be used to quickly screen single to multiple genes for TE density differences in a new reference genome. The inclusion of CFTR is simply to provide an additional example where a TE variant near a target gene is associated with a disease trait in humans (e.g. cystic fibrosis). This aspect of the tool can be applied to any trait in any system (e.g. TE insertion associated with variation in a key target trait in any crop).

### Gene ontology enrichment analysis of TE-Dense genes

TE Density data may be leveraged to create a list of genes suitable for gene ontology (GO) enrichment analyses. A percentile cutoff can easily be used to generate a list of genes for analysis. Here, considering all genes in the *Oryza sativa* genome, we selected genes whose 1KB upstream LTR element density was within the 99th percentile. Calculating this percentile generated a TE density cutoff value of 0.863 and yielded a list of 379 genes (see Supplemental fileLTR_file_1000.tsv).

Next, we passed our list through PANTHER’s Overrepresentation Test (Version 16.0) using the Panther GO-Slim Biological Process annotation data set. Table 3 displays the output of the analysis, revealing that metabolic processes are underrepresented in this set of TE-dense genes. This suggests that LTR elements were selectively lost from the upstream regions of genes belonging to those listed functional classes. We also screened a random subset of the bottom 1% of TE-dense genes and found no significant enrichment. Functional characteristics of genes have been hypothesized and shown as factors affecting the selection of TE insertions near genes [10, 14, 30, 59, 69], and the TE Density tool offers a new, a priori way to investigate this relationship.

**Table 3:** Output from Panther GO-Slim Biological Process Analysis

PANTHER GO-Slim Biological Process	Fold Enrichment	FDR
Unclassified (UNCLASSIFIED)	1.11	0.00552
biological process (GO:0008150)	0.49	0.00276
cellular process (GO:0009987)	0.48	0.00687
organic substance metabolic process (GO:0071704)	0.46	0.0279
metabolic process (GO:0008152)	0.46	0.0175
primary metabolic process (GO:0044238)	0.44	0.0283
nitrogen compound metabolic process (GO:0006807)	0.42	0.0282
cellular metabolic process (GO:0044237)	0.42	0.0157

## Discussion

One of the main strengths and limitations of the TE density tool is its reliance on gene and TE annotation files. The organization of text data in annotation files can be rather variable, thus importing them to use in the pipeline requires some basic pre-processing to acquire the correct gene and TE identities. In order to make this process easier, we provide example scripts and guides in the source code and project web-page. Another drawback of using annotation files is that the boundaries of TEs and genes in the annotation files can differ depending on the type and version of software used to generate each respective annotation; this may impact the ability to draw comparisons between systems that use different annotation software.

However, this is also a strength as it allows users to use annotation files of their choice as inputs to TE Density, affording a degree of flexibility. In order to simplify our calculations, we defined a gene as the inclusive space from the start position of the first exon to the stop position of the last exon. This disables the ability to distinguish between a TE that is truly intronic or one that overlaps with an exon, thus we use the term “intragenic” to describe TEs found within the previously described boundary.

One difficulty in interpreting TE Density results is that the relative abundance, length, and genomic distributions of TEs in the given genome can impact the calculation of TE density. For example, LINE elements are quite uncommon in plant genomes; a plant genome would likely show an overwhelming proportion of genes with a value of 0 LINE TE density across all combinations of windows and positions relative to genes. This could easily lead to the conclusion that LINE elements are not tolerated near genes but that trend is much better explained by the relative paucity of LINE elements in the genome. Interpreting the density values of short TEs (MITEs, SINES, and others) with longer TEs such as LTRs is difficult. For example, one genomic region with several SINEs could produce the same density value as a region possessing one LTR. Additionally, genomic distributions and other properties of TEs can differ at the family level; this can reduce our ability to draw general trends from the TE groupings used here, Order and Superfamily, as they can aggregate potentially disparate TE families.

This tool offers an improvement over previous assessments of TE presence by utilizing an algorithm that calculates TE density for *all* genes in the genome, for *all* TE types, upstream, intragenically, and downstream, over a set of user-defined measurement windows. The tool generates an output array for each pseudomolecule of input data, calculating TE density values for the combination of TE (*superfamily* ∥ *order*)×(*left* ∥ *intra* ∥ *right*), with respect to a window length and a specific gene. Previous attempts at quantifying TE presence failed to provide or provided limited source code, documentation, and test verification of software.

## Conclusion

The TE Density tool represents a new, reproducible way to quantify TE presence surrounding genes. The tool’s data can be used to examine TE presence genome-wide, TE presence between genomes, and TE presence at the individual gene scale. The data can be used as a screen to examine changes in TE presence, or as part of a larger analysis incorporating other datasets such as methylation or gene expression data. The analysis scripts used to create the figures in this article are also provided with the source code, and were designed with community-usage in mind so that others may build off of what is presented here.

## Availability and requirements

**Project name** TE Density

**Project home page**https://github.com/sjteresi/TE_Density.

**Operating System** Platform independent

**Programming language** Python

**Other requirements** Python 3.8.0, h5py 2.10.0, numpy 1.20.2, pandas 1.0.5, see requirements directory in project GitHub repository for more complete list of minor Python packages

**License** GNU GPL 3.0

## Supplementary Information

**Additional file 1** Supplementary figures and table.

**Additional file 2** Supplementary materials.

## Data Availability

The datasets generated during the current study are available at https://datadryad.org/stash/share/mFjpHlP53Y-BUP4nKI0LQGVmLkXevNatnz8MLlK36zw.

## Abbreviations

TE: Transposable element
HDF5: Hierarchical Data Format Version 5: A file format and software suite useful in storing complex heterogenous data such as data matrices with very high dimensions
