# Supplementary material for: TE Density: a tool to investigate the biology of transposable elements
Source: Mob DNA. 2022 Apr 12;13:11. doi: 10.1186/s13100-022-00264-4 (PMC9004194; doi:10.1186/s13100-022-00264-4)
Supplement: Supplementary file 1 — Additional file 1 Supplementary figures and table. [file 13100_2022_264_MOESM1_ESM.pdf]

# 1 SUPPLEMENTAL FIGURES AND TABLE

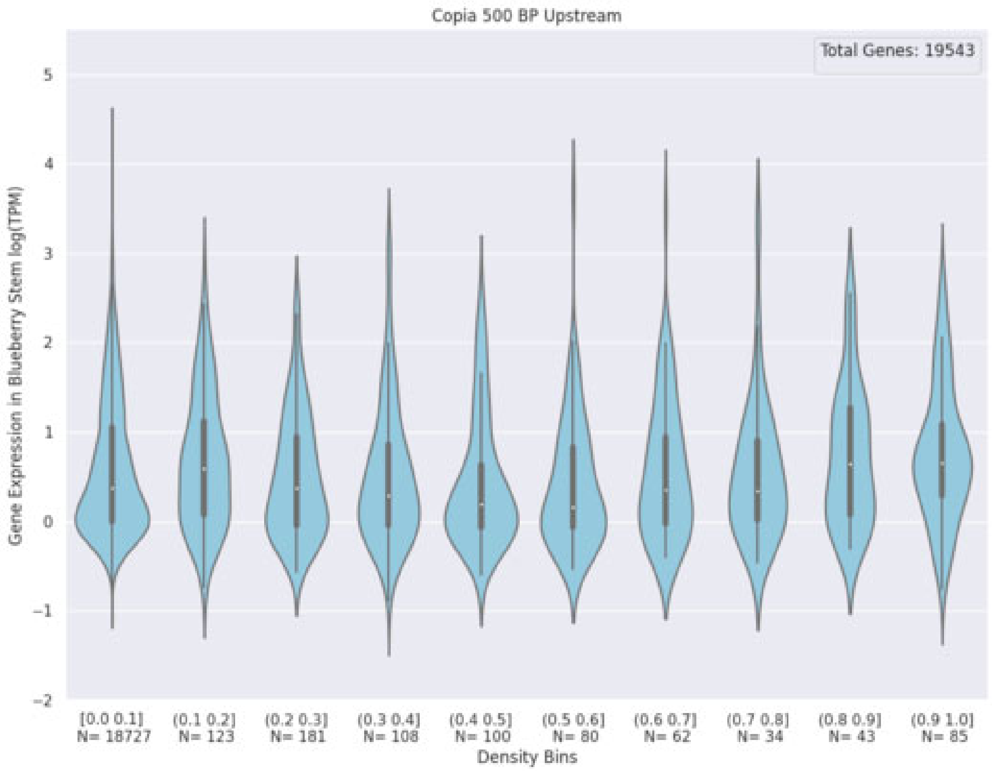


**Figure S1.** Violin Plots of TE Density vs Gene Expression: Density values are derived from the Copia TE grouping for the 500 BP window downstream of each gene. Underneath each violin plot is the interval of TE density values that bins the genes being plotted. Underneath each density bin is N, the number of genes for that given bin. Lowly expressed genes, genes with less than 0.1 TPM, were excluded from the plot. The solid dark band inside each violin represents the interquartile range (IQR) of the expression values. The white dot inside the IQR represents the median. The “whiskers” extend 1.5x past the IQR in both directions.

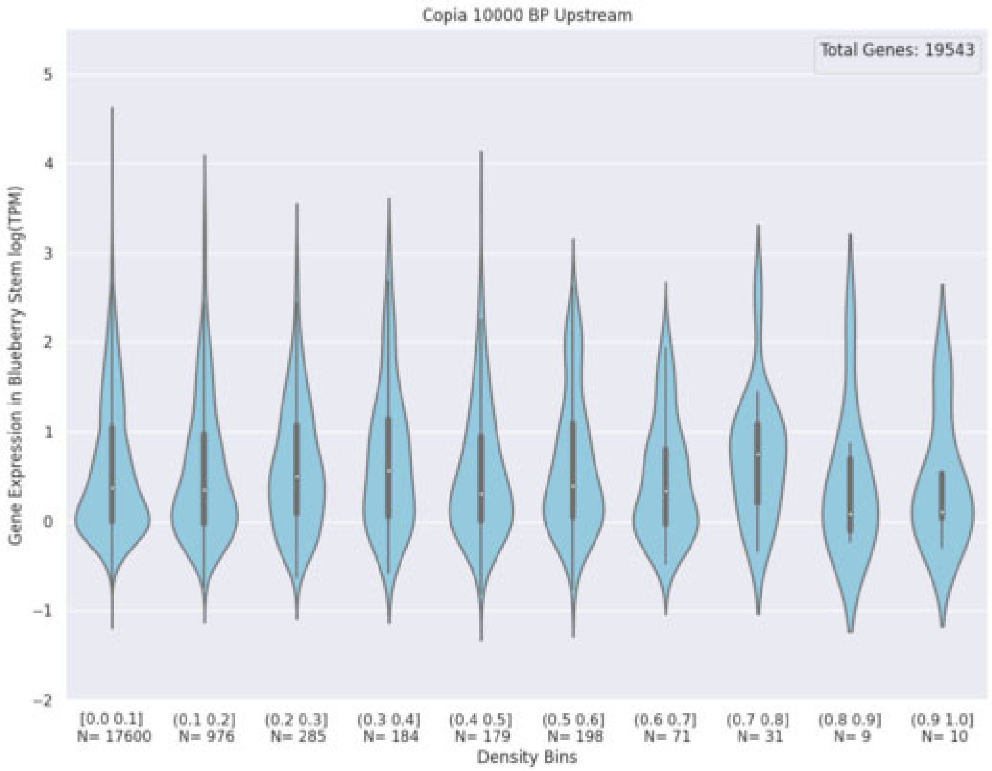


**Figure S2.** Violin Plots of TE Density vs Gene Expression: Density values are derived from the Copia TE grouping for the 10000 BP window downstream of each gene. Underneath each violin plot is the interval of TE density values that bins the genes being plotted. Underneath each density bin is N, the number of genes for that given bin. Lowly expressed genes, genes with less than 0.1 TPM, were excluded from the plot. The solid dark band inside each violin represents the interquartile range (IQR) of the expression values. The white dot inside the IQR represents the median. The “whiskers” extend 1.5x past the IQR in both directions.

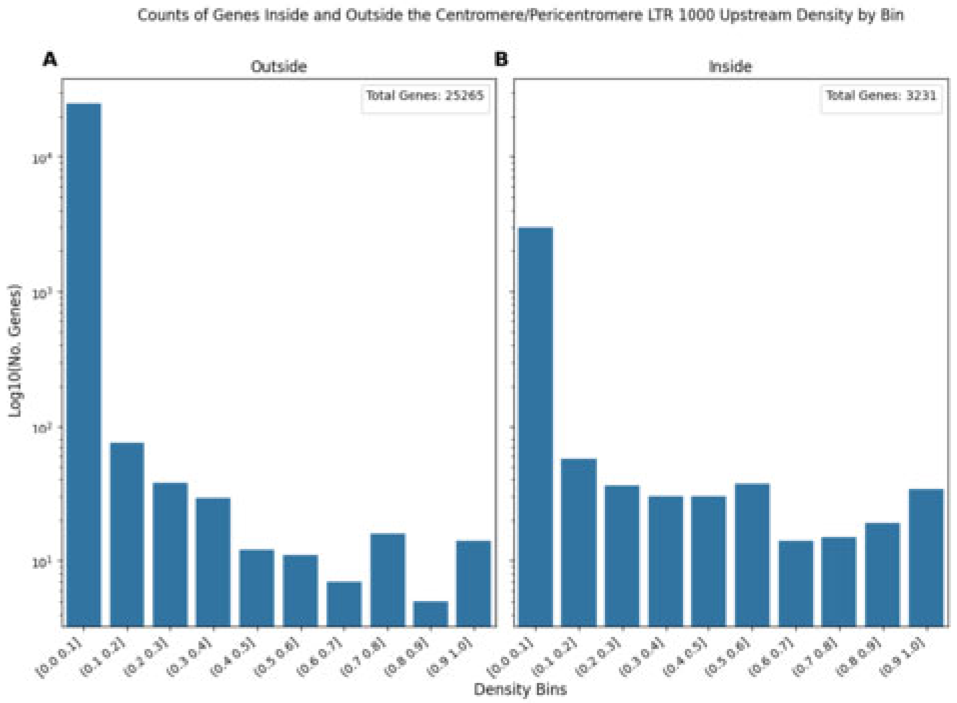


**Figure S3.** Bar plots of the number of genes in a given TE density bin as TE density increases, genes are grouped by status of belonging to the centromere/pericentromere or not. TE density was assessed for the LTR TE upstream of all genes. Subplot **A** represents the genes that *do not* belong to the centromere or pericentromere. Subplot **B** represents the genes that *do* belong the pericentromere or centromere.

| Top 5 TE Orders | | | | | |
|---|---|---|---|---|---|
| **Upstream:** | | | | | |
| Identity | NA | NA | Unknown | SINE | Total TE Density |
| Density | 0 | 0 | 0.045 | 0.054 | 0.103 |
| **Intragenic:** | | | | | |
| Identity | LTR | TIR | SINE | LINE | Total TE Density |
| Value | 0.023 | 0.037 | 0.110 | 0.258 | 0.429 |
| **Downstream:** | | | | | |
| Identity | NA | NA | NA | NA | NA |
| Value | 0 | 0 | 0 | 0 | 0 |
| **Top 5 TE Superfamilies** | | | | | |
| **Upstream:** | | | | | |
| Identity | NA | NA | Unknown | Alu | Total TE Density |
| Density | NA | NA | 0.049 | 0.0540 | 0.103 |
| **Intragenic:** | | | | | |
| Identity | MIR | L2 | Alu | L1 | Total TE Density |
| Value | 0.036 | 0.052 | 0.074 | 0.201 | 0.429 |
| **Downstream:** | | | | | |
| Identity | NA | NA | NA | NA | NA |
| Value | 0 | 0 | 0 | 0 | 0 |

**Table 1.** Table of greatest TE density values by TE type for the CFTR gene in a 1 KB window upstream and downstream.
